# Interpreting declines in HIV test positivity: an analysis of routine data from Zimbabwe's national sex work programme, 2009–2019

**DOI:** 10.1002/jia2.25943

**Published:** 2022-06-30

**Authors:** Harriet S. Jones, Bernadette Hensen, Sithembile Musemburi, Lilian Chinyanganya, Albert Takaruza, Sungai T. Chabata, Primrose Matambanadzo, Brian Rice, Frances M. Cowan, James R. Hargreaves

**Affiliations:** ^1^ Faculty of Public Health and Policy, London School of Hygiene and Tropical Medicine London UK; ^2^ Faculty of Epidemiology & Population Health, London School of Hygiene & Tropical Medicine London UK; ^3^ Centre for Sexual Health and HIV/AIDS Research (CeSHHAR) Zimbabwe Harare Zimbabwe; ^4^ Faculty of Global Health, Liverpool School of Tropical Medicine Liverpool UK

**Keywords:** Africa, HIV epidemiology, HIV prevention, key and vulnerable populations, sex workers, testing

## Abstract

**Introduction:**

Early diagnosis of HIV is critical for epidemic control. To achieve this, successful testing programmes are essential and test positivity is often used as a marker of their performance. The aim of this study was to analyse trends and predictors of HIV test positivity over time and explore how an understanding of seroconversion rates could build on our interpretation of this indicator among female sex workers in Zimbabwe.

**Methods:**

We analysed HIV test data from Zimbabwe's nationally scaled sex work programme between 2009 and 2019. We defined test positivity as the proportion of all tests that were HIV positive and measured new diagnoses by estimating seroconversion rates among women with repeat tests, defined as an HIV‐positive test after at least one HIV‐negative test in the programme. We used logistic regression to analyse test positivity over three time‐periods: 2009–2013, 2014–2017 and 2018–2019, adjusting for potential confounding by demographic factors and the mediating effects of time since last HIV test. We calculated the seroconversion rates for the same time‐periods.

**Results:**

During the 10‐year study period, 54,503 tests were recorded in 39,462 women. Between 2009 and 2013, 18% of tests were among women who reported testing in the previous 6 months. By 2018–2019, this had increased to 57%. Between 2018 and 2019, test positivity was 9.6%, compared to 47.9% for 2009–2013 (aOR 6.08 95% CI 5.52–6.70) and 18.8% for 2014–2017 (aOR 2.17 95% CI 2.06–2.28). Adjusting for time since last test reduced effect estimates for 2009–2013 (aOR 4.03 95% CI 3.64–4.45) and 2014–2017 (aOR 1.97 95% CI 1.86–2.09) compared to 2018–2019. Among 7573 women with an initial HIV‐negative test in the programme and at least one subsequent test, 464 tested HIV positive at a rate of 3.9 per 100 pyar (95% CI 3.5–4.2).

**Conclusions:**

Test positivity decreased among women testing through the programme over time, while seroconversion rates remained high. These declines were partly driven by changes in individual testing history, reflecting comprehensive coverage of testing services and greater knowledge of HIV status, but not necessarily declining rates of seroconversion. Understanding testing history and monitoring new HIV infections from repeat tests could strengthen the interpretation of test positivity and provide a better understanding of programme performance.

## 1 INTRODUCTION

Early diagnosis of HIV is critical for epidemic control. Female sex workers (FSW) in sub‐Saharan Africa are at greater risk of HIV infection than other women of reproductive age, and sex work an important driver of HIV transmission [1, 2, 3]. Yet, globally, the proportion of FSW diagnosed fell short of UNAIDS 2020 targets of 90% [4]. In Zimbabwe, UNAIDS report 75.4% of FSW knew their HIV‐positive status in 2020, compared to 96% of all adult women [5]. Annual HIV testing is recommended for FSW in all settings, and testing every 3–6 months if indicated by individual risk [6]. Successful testing strategies are fundamental for identifying individuals with HIV, but where incidence remains high, even intensive strategies may fail to identify enough cases to reach the UNAIDS 2030 target of 95% of those with HIV knowing their HIV‐positive status [7].

The performance of testing programmes is often monitored using HIV test yield or test positivity, defined as the proportion of tests that are HIV positive [8, 9]. Funding constraints have made it necessary for programmes to balance resource efficiencies with identifying a decreasing proportion of individuals with undiagnosed HIV [9, 10]. Test positivity has been used to evaluate differentiated HIV testing approaches being implemented to achieve this, such as community‐based testing, self‐testing, index‐testing and partner notification [11, 12, 13, 14, 15, 16]. Individual testing history and repeat testing among HIV‐negative individuals [17, 18] will play a role in test positivity but have less frequently been explored. Test positivity will be influenced by all of these factors, as well as HIV incidence and prevalence, testing coverage and re‐diagnosis [16, 17, 19, 20], and should be interpreted in the context of these complexities to understand programme effectiveness and gauge progress towards global targets.

In Zimbabwe, the Sisters with a Voice programme (Sisters) offers HIV testing, alongside other sexual and reproductive health services for FSW nationally. In 2017, Sisters reached 57% of the estimated 40,000 FSW in Zimbabwe with clinical services [3]. Since 2009, the programme has collected routine service delivery data, providing a unique opportunity to explore long‐term trends in HIV testing. The aim of this analysis was to understand trends in HIV test positivity between 2009 and 2019, and identify the individual and service delivery factors influencing these. We further sought to identify how trends in seroconversion among repeat testers could build on our interpretation of test positivity as an indicator of programme performance.

## 2 METHODS

### 2.1 Study setting

The Sisters programme delivers free sexual and reproductive health services through static and mobile sites across Zimbabwe to women aged ≥16 years self‐identifying as selling sex [21]. HIV testing is offered at a first clinic visit if an HIV negative or unknown HIV status is reported. In line with national guidance, women revisiting a clinic are offered an HIV test if they have not tested within the previous 6 months. Since 2014, Determine HIV‐1/2 has been used as a first screening test with SD Bioline HIV‐1/2 to confirm HIV‐positive results.

**Figure 1:**
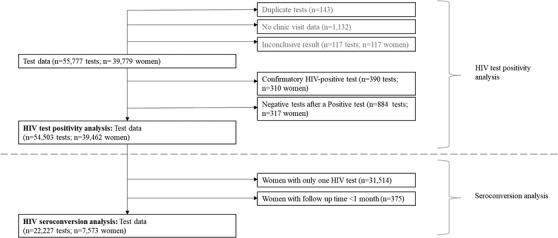
Flow diagram of women included in HIV test positivity and seroconversion analysis.

At each visit, a woman is seen by clinic staff and data are collected on demographic variables (first visit only), the reason for her visit, self‐report STI and HIV test and test result history, a sexual risk behaviour history, the services provided at that visit, and the results of any syndromic STI diagnosis and HIV test. Data are electronically kept and centrally held for each woman, linked by a unique identification number and a Sisters number assigned at first visit. Women are subsequently identified by their Sisters number or unique identifying information if this is not known. Further checks are carried out during regular data syncing to ensure that multiple records do not exist for the same woman. HIV test results, clinical and demographic data are held in separate databases, which were merged for this analysis, matching records on Sisters number and clinic visit date. We excluded tests if results were inconclusive, duplicated (defined as a second test within 7 days of a previous programme test) or confirming an existing HIV‐positive result within the programme. We excluded women from our analysis if they had an HIV‐negative test after an HIV‐positive test as we could not guarantee data accuracy (Figure 1).

### 2.2 Measures

Our main outcome was HIV test positivity, defined as the proportion of all HIV tests delivered by the programme that were HIV positive. We then restricted our analysis to women with >1 test to explore trends in new HIV diagnoses in the programme by estimating seroconversion rates. We defined seroconversion as an HIV‐positive test after at least one HIV‐negative test at a Sisters clinic.

Our main exposure was calendar time. We analysed changes over three pre‐specified periods of varying programme implementation. Our first period covered early implementation from 2009 to 2013. Five Sisters sites were established in areas known for high numbers of sex workers, but there were delays in funding continuation for much of 2012 and disruption of services in 2013 due to elections (static sites: 1 in 2009, 3 between 2010 and 2012, 6 in 2013; outreach sites: 4 between 2009 and 2010; 10 between 2010 and 2013, 30 in 2013). The number of clinic sites increased to 36 by the end of 2017 (static: 6 between 2014 and 2017, outreach: 30 between 2014 and 2017), and outreach had gone from once every 2 weeks to once a week, representing significant programme expansion, financial input and increased recruitment of FSW through intensified peer outreach. The third period, 2018–2019, represents a more established programme with 57 clinic sites (12 static) but funding disruptions, forcing periodic clinic closures [3].

We analysed demographic (age, education and marital status), self‐report HIV testing history (time since last test at a Sisters clinic or externally) and HIV status and clinic visit (clinic location and type, reason for a clinic visit and STI diagnoses) variables. Age was calculated from date of birth to reflect age at the date of each clinic visit, and categorized as <25 and ≥25 years old. A self‐reported test history, including date and result of last test, was also collected at each visit and categorized as never tested, tested in the previous 6 months, 6–12 months or >12 months. To address missing or implausible data on testing history (e.g. when a date was in the future), we used self‐report or programme test data from earlier visits to complete records where possible.

### 2.3 Analysis

We described women visiting and HIV testing in the programme and plotted test positivity and testing history by year quarter to understand trends over time. Using logistic regression, we estimated the crude association between time‐period and test positivity, and explored potential associations with FSW characteristics (demographics, test history and clinic visit information) to identify predictors of positivity. Our models included time‐period as an interaction term to understand if associations varied over time.

We adjusted our test positivity and time‐period logistic regression model for FSW characteristics to explore confounding. We analysed the mediating role of HIV testing history in the relationship between time‐period and test positivity by further adjusting for time of last HIV test. Our models included robust standard errors to account for clustering by site and repeat tests on the same women. We conducted a sensitivity analysis with calendar year as our exposure to assess the impact of our time‐period assumptions on our findings. Our models included the maximum number of records available at each stage to obtain the least biased estimate. We conducted a final analysis using the subset of data included in our fully adjusted model to understand if this approach had biased our results.

We estimated HIV seroconversion among women returning to Sisters clinics for an HIV test using an approach previously applied to a subset of our data [22]. We established a retrospective cohort of women to include in our analysis. Women were eligible if they had more than one HIV test at a Sisters clinic, their first test was HIV negative and their last HIV test with the programme was more than 1 month after their first. Date of entry was a woman's first HIV test at a Sisters clinic. Date of seroconversion was estimated at the midpoint between a woman's last HIV‐negative test and her HIV‐positive test. Exit date was either the estimated date of seroconversion or last HIV‐negative test (if no HIV‐positive result). We used lexis expansion to split our data by time‐period and calculated seroconversion rates for each using robust standard errors to account for clustering by site. Lastly, we compared our findings with those previously published from these data by looking at the seroconversion rate between September 2009 and May 2013.

### 2.4 Ethics

Ethical approval was obtained from the London School of Hygiene and Tropical Medicine (16543) and the Medical Research Council of Zimbabwe (MRCZ/A/2624). All data in this analysis were collected as part of routine clinical care and, therefore, consent was not obtained. Data were de‐identified and anonymized before databases were shared for analysis.

## 3 RESULTS

Between September 2009 and December 2019, 86,197 women made 254,653 visits to a Sisters clinic. Half of all women visited once (44,852/86,197; 52.0%), 17.6% (15,186/86,197) visited twice, 17.9% (15,468/86,197) had between 3 and 5 visits and 12.4% (10,691/86,197) >5. At first visit, median age was 28 years (IQR 23–34), 68.7% (59,245/86,197) reached secondary education and 60.9% (52,491/86,197) were divorced. Just under half of all clinic visits were attended by women self‐reporting an HIV‐positive status (Table 1).

**Table 1:** Characteristics of women visiting and HIV testing at Sisters clinics between 2009 and 2019 by time‐period

	All clinic visits and HIV tests 2009–2019		Time‐period 1 2009–2013		Time‐period 2 2014–2017		Time‐period 3 2018–2019	
	Total clinic visits	Total HIV tests	Clinic visits *N* (col%)	HIV tests *N* (col%)	Clinic visits *N* (col%)	HIV tests *N* (col%)	Clinic visits *N* (col%)	HIV tests *N* (col%)
Total (row %)	*n* = 254,653	*n* = 54,503	*n* = 36,426	*n* = 4039 (11.1)	*n* = 139,199	*n* = 23,440 (16.8)	*n* = 79,028	*n* = 27,024 (34.2)
**Demographic**								
Age (at first clinic visit)								
<25	57,659 (23.4)	19,343 (37.0)	6595 (18.2)	1047 (26.0)	31,658 (23.5)	8281 (36.8)	19,406 (25.8)	10,015 (39.0)
25+	188,422 (76.6)	32,885 (63.0)	29,693 (81.8)	2980 (74.0)	102,986 (76.5)	14,246 (63.2)	55,743 (74.2)	15,659 (61.0)
*missing*	*8572*	*2275*	*138*	*12*	*4555*	*913*	*3879*	*1350*
Education								
None	1635 (0.7)	295 (0.6)	34 (0.2)	4 (0.2)	1137 (0.9)	186 (0.8)	464 (0.6)	105 (0.4)
Primary	51,364 (23.3)	9642 (19.3)	5371 (27.4)	620 (24.6)	31,821 (24.9)	4900 (22.2)	14,172 (19.5)	4122 (16.3)
Secondary	165,565 (75.2)	39,267 (78.7)	14,076 (71.9)	1883 (74.7)	94,414 (73.7)	16,795 (76.2)	57,075 (78.5)	20,589 (81.3)
Tertiary	1754 (0.8)	687 (1.4)	96 (0.5)	14 (0.6)	697 (0.5)	168 (0.8)	961 (1.3)	505 (2.0)
*missing*	*34,335*	*4612*	*16,849*	*1518*	*11,130*	*1391*	*6356*	*1703*
Marital status								
Currently married	4955 (2.2)	1494 (2.9)	521 (1.4)	64 (1.6)	2608 (2.0)	583 (2.6)	1826 (2.5)	847 (3.3)
Divorced	156,536 (64.0)	32,031 (61.7)	21,372 (59.1)	2473 (61.7)	88,557 (66.1)	14,667 (65.4)	46,607 (62.5)	14,891 (58.4)
Never married	47,634 (19.5)	14,415 (27.8)	5892 (16.3)	748 (18.7)	22,690 (16.9)	5071 (22.6)	19,052 (25.6)	8596 (33.7)
Separated	2985 (1.2)	360 (0.7)	930 (2.6)	108 (2.7)	1726 (1.3)	205 (0.9)	329 (0.4)	47 (0.2)
Widowed	32,614 (13.3)	3622 (7.0)	7457 (20.6)	618 (15.4)	18,419 (13.7)	1891 (8.4)	6738 (9.0)	1113 (4.4)
*missing*	*9929*	*2581*	*254*	*28*	*5199*	*1023*	*4476*	*1530*
**Clinic site**								
Location								
Urban	196,473 (77.2)	37,135 (68.1)	28,795 (79.1)	2969 (73.5)	100,538 (72.2)	12,882 (55.0)	67,140 (85.0)	21,284 (78.8)
Rural	58,180 (22.9)	17,368 (31.9)	7631 (21.0)	1070 (26.5)	38,661 (27.8)	10,558 (45.0)	11,888 (15.0)	5740 (21.2)
Type								
Static	149,740 (58.8)	45,021 (82.6)	22,469 (81.7)	3445 (85.3)	68,284 (49.1)	23,880 (88.4)	58,987 (74.6)	17,696 (75.5)
Mobile	104,913 (41.2)	9482 (17.4)	13,957 (38.3)	594 (14.7)	70,915 (51.0)	3144 (11.6)	20,041 (25.4)	5744 (24.5)
**HIV testing history**								
Time since last HIV test								
Never tested	12,051 (4.9)	5974 (11.5)	5381 (15.3)	1563 (40.5)	4162 (3.0)	2309 (10.2)	2508 (3.3)	2102 (8.3)
Tested >12 months ago	82,030 (33.1)	9088 (17.5)	13,338 (37.8)	882 (22.9)	45,634 (33.4)	4514 (19.9)	23,058 (30.4)	3692 (14.5)
Tested 6–12 months ago	35,898 (14.5)	10,808 (20.8)	5552 (15.7)	711 (18.4)	19,621 (14.4)	4844 (21.3)	10,725 (14.1)	5253 (20.6)
Tested <6 months ago	117,913 (47.6)	26,168 (50.3)	11,002 (31.2)	702 (18.2)	67,296 (49.2)	11,031 (48.6)	39,615 (52.2)	14,435 (56.7)
*missing*	6761	2465	1153	181	2486	742	3122	1542
Self‐report HIV status								
HIV negative	127,785 (54.1)	46,241 (97.1)	11,088 (39.3)	1939 (92.2)	72,072 (54.5)	20,136 (97.0)	44,625 (59.1)	24,166 (97.6)
HIV positive	108,322 (45.9)	1370 (2.9)	17,105 (60.7)	164 (7.8)	60,286 (45.5)	611 (3.0)	30,931 (40.9)	595 (2.4)
*missing*	*18,546*	*6892*	*8233*	*1936*	*6841*	*2693*	*3472*	*2263*
**Sisters clinic engagement**								
Clinic visits								
First visit	168,456 (66.2)	31,288 (57.4)	22,568 (62.0)	2514 (62.2)	92,481 (66.4)	13,470 (57.5)	53,407 (67.6)	15,304 (56.6)
Repeat visit	86,197 (33.9)	23,215 (42.6)	13,858 (38.0)	1525 (37.8)	46,718 (33.6)	9970 (42.5)	25,621 (32.4)	11,720 (43.4)
STI diagnosed at clinic visit								
No	159,619 (62.7)	35,556 (65.2)	22,157 (60.8)	2212 (54.8)	80,034 (57.5)	13,794 (58.9)	57,428 (72.7)	19,550 (72.3)
Yes	95,034 (37.3)	18,947 (34.8)	14,269 (39.2)	1827 (45.2)	59,165 (42.5)	9646 (41.2)	21,600 (27.3)	7474 (27.7)
Visit for family planning								
No	212,160 (83.3)	45,053 (82.7)	33,236 (91.2)	3616 (89.5)	117,350 (84.3)	19,997 (85.3)	61,574 (77.9)	21,440 (79.3)
Yes	42,493 (16.7)	9450 (17.3)	3190 (8.8)	423 (10.5)	21,849 (15.7)	3443 (14.7)	17,454 (22.1)	5584 (20.7)
Testing delivery								
First programme test	39,462 (72.4)	39,462 (72.4)	3560 (88.1)	3560 (88.1)	17,992 (76.7)	17,992 (76.7)	17,910 (66.3)	17,910 (66.3)
Repeat programme test	15,041 (27.6)	15,041 (27.6)	479 (11.9)	479 (11.9)	5448 (23.2)	5448 (23.2)	9114 (33.7)	9114 (33.7)

**Figure 2:**
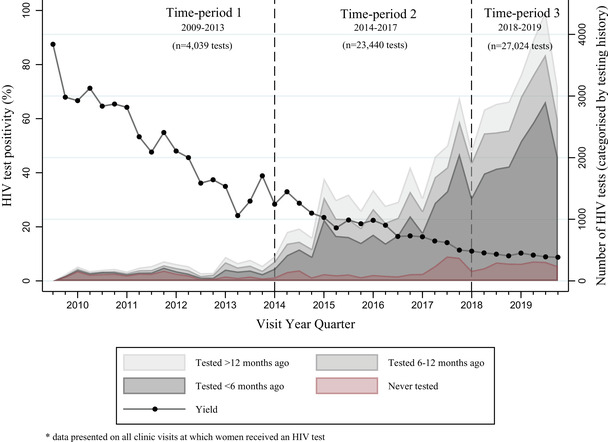
HIV test positivity and testing coverage at Sisters clinics in Zimbabwe between 2009 and 2019.

During the study period, 55,777 HIV tests were conducted and data on 54,503 tests among 39,462 women included in the analysis (Figure 1). Overall, missing data on demographic and testing history variables did not exceed 10%, with small variations in the proportion missing between HIV‐positive and HIV‐negative tests, and slightly more between time‐periods. Tests among women reporting having never tested fell from 38.7% (1563/4039) between 2009 and 2013 to 11.3% (2102/27,024) between 2018 and 2019. In later time‐periods, most tests were among women self‐reporting or having tested at a Sisters clinic in the previous 6 months. Between 2018 and 2019, this was 56.7% (14,453/27,024), compared to 17% (702/4039) between 2009 and 2013 (Figure 2). Over time, an increasing percentage of tests were among women <25 years old, from 26.0% (1047/4039) between 2009 and 2013 to 39.0% (10,015/27,024) between 2018 and 2019. A small percentage of tests (1370/54,503, 2.9%) were among women self‐reporting an HIV‐positive status (Table 1).

Between 2009 and 2019, 16.4% (8959/54,503) of programme tests were HIV positive. Test positivity decreased from 47.9% (1934/4039) between 2009 and 2013, to 18.8% between 2014 and 2017 (4417/23,440; OR 2.2 95% CI 2.1–2.3 *p*<0.001) and 9.7% between 2018 and 2019 (2608/27,024; OR 8.6 95% CI 7.9–9.3 *p*<0.001) (Table 3). In all time‐periods, test positivity was higher among women ≥25 years old than <25 years old (OR 1.50 95% CI 1.4–1.7 *p*<0.001). However, test positivity declined more steadily among women <25 years old who made up an increasing proportion of women testing HIV positive over time, from 20.8% (403/1934) between 2009 and 2013 to 31.4% (819/2608) between 2018 and 2019. Test positivity was higher among women with primary than secondary education (OR 1.33 95% CI 1.22–1.46), and those diagnosed with an STI at a Sisters clinic compared to those who were not (OR 1.91 95% CI 1.75–2.09 *p*<0.001). For 2009–2013, test positivity was higher among women visiting for family planning than those who visited for other reasons (OR 2.2 95% CI 1.73–2.85), but the opposite in later time‐periods. Test positivity was also higher at first‐time programme tests than repeat tests at a Sisters clinic (OR 7.88 95% CI 6.62–9.38 *p*<0.001) (Table 2).

**Table 2:** Univariable and stratified logistic regression analysis of time, demographic, HIV testing history and clinic and service engagement factors with HIV test positivity

					**HIV‐positive tests**									
	**All HIV tests between 2009 and 2019**				**2009–2013**			**2014–2017**			**2018–2019**			**Interaction**
	**Total tests**	**HIV‐positive tests (row%)**	**OR (95% CI)**	***p*‐value**	**Total**	**HIV‐positive tests (row%)**	**OR (95% CI)**	**Total**	**HIV‐positive tests (row%)**	**OR (95% CI)**	**Total**	**HIV‐positive tests (row%)**	**OR (95% CI)**	***p*‐value (Wald)**
All HIV tests	54,503	8959 (16.4)	2.2 (2.1–2.3)		4039	1934 (47.9)		23,440	4417 (18.8)		27,024	2608 (9.7)		
**Demographic**														
Age (at first clinic visit)														
<25	19,343	2560 (13.2)	1 (baseline)		1047	403 (38.5)	1 (baseline)	8281	1338 (16.2)	1 (baseline)	10,015	819 (8.2)	1 (baseline)	0.10
25+	32,885	6139 (18.7)	1.50 (1.37–1.65)	<0.001	2980	1522 (51.1)	1.67 (1.39–2.00)	14,246	2966 (20.8)	1.36 (1.19–1.57)	15,659	1651 (10.5)	1.32 (1.17–1.49)	
*missing*	*2275*	*260 (11.4)*			12	9 (75)		913	113 (12.4)		1350	138 (10.2)		
Education														
None	295	60 (20.3)	1.47 (0.94–2.29)	<0.001	4	2 (50.0)	1.42 (0.24–9.46)	186	43 (23.1)	1.33 (0.81–2.18)	105	15 (14.3)	1.57 (0.69–3.56)	0.004
Primary	9642	1814 (18.8)	1.33 (1.22–1.46)		620	287 (46.3)	1.31 (1.19–1.43)	4900	1088 (22.2)	1.27 (1.13–1.42)	4122	439 (10.6)	1.13 (1.00–1.27)	
Secondary	39,267	5814 (14.8)	1 (baseline)		1883	749 (39.8)	1 (baseline)	16,795	3092 (18.4)	1 (baseline)	20,589	1973 (9.6)	1 (baseline)	
Tertiary	687	41 (6.0)	0.37 (0.25–0.52)		14	5 (35.7)	0.84 (0.21–3.40)	168	13 (7.7)	0.37 (0.20–0.69)	505	23 (4.6)	0.45 (0.32–0.64)	
*missing*	*4612*	*1230 (26.7)*			*1518*	*891 (58.7)*		*1391*	*181 (13.0)*		*1703*	*158 (9.3)*		
Marital status														
Currently married	1494	169 (11.3)	0.60 (0.50–0.71)	<0.001	64	23 (35.9)	0.63 (0.35–1.15)	583	73 (12.5)	0.56 (0.38–0.83)	847	73 (8.6)	0.85 (0.51–1.41)	<0.001
Divorced	32,031	5621 (17.6)	1 (baseline)		2473	1162 (47.0)	1 (baseline)	14,667	2968 (20.2)	1 (baseline)	14,891	1491 (10.0)	1 (baseline)	
Never married	14,415	1794 (12.5)	0.67 (0.63–0.71)		748	302 (40.4)	0.76 (0.67–0.87)	5071	736 (14.5)	0.67 (0.56–0.79)	8596	756 (8.8)	0.87 (0.76–0.99)	
Separated	360	66 (18.3)	1.05 (0.77–1.45)		108	31 (28.7)	0.45 (0.32–0.64)	205	33 (16.1)	0.76 (0.47–1.22)	47	2 (4.3)	0.40 (0.09–1.73)	
Widowed	3622	1018 (28.1)	1.84 (1.68–2.01)		618	397 (64.2)	2.03 (1.76–2.33)	1891	481 (25.4)	0.35 (1.11–1.62)	1113	140 (12.6)	1.29 (1.05–1.59)	
*missing*	*2581*	*291 (11.3)*			*28*	*19 (67.9)*		*1023*	*126 (12.3)*		*1530*	*146 (9.5)*		
**Clinic site**														
Location														
Urban	45,021	7425 (16.5)	1 (baseline)		3445	1629 (47.3)	1 (baseline)	17,696	3408 (19.3)	1 (baseline)	23,880	2388 (10.0)	1 (baseline)	0.007
Rural	9482	1534 (16.2)	0.98 (0.72–1.33)	0.88	594	305 (51.4)	1.18 (0.70–1.97)	5744	1009 (17.6)	0.89 (0.68–1.18)	3144	220 (7.0)	0.68 (0.52–0.88)	
Type														
Static	37,135	6007 (16.2)	1 (baseline)		2969	1455 (49.0)	1 (baseline)	12,882	2425 (18.8)	1 (baseline)	21,284	2127 (10.0)	1 (baseline)	0.23
Mobile	17,368	2952 (17.0)	1.06 (0.74–1.53)	0.75	1070	479 (44.8)	0.84 (0.44–1.60)	10,558	1992 (18.9)	1.00 (0.75–1.35)	5740	481 (8.4)	0.82 (0.62–1.09)	
**HIV testing history**														
Time since last HIV test														
Never tested	5974	2067 (34.6)	1 (baseline)	<0.001	1563	1125 (72.0)	1 (baseline)	2309	705 (30.5)	1 (baseline)	2102	237 (11.3)	1 (baseline)	<0.001
Tested >12 months ago	9088	2705 (29.8)	0.80 (0.75–0.86)		882	396 (44.9)	0.32 (0.27–0.38)	4514	1556 (34.5)	1.20 (1.08–1.33)	3692	753 (20.4)	2.02 (1.72–2.36)	
Tested 6–12 months ago	10,808	1531 (17.2)	0.31 (0.29–0.34)		711	143 (20.1)	0.10 (0.08–0.12)	4844	859 (17.7)	0.49 (0.44–0.55)	5253	529 (10.1)	0.88 (0.75–1.04)	
Tested <6 months ago	26,168	2187 (8.4)	0.17 (0.16–0.18)		702	160 (22.8)	0.11 (0.09–0.14)	11,031	1127 (10.2)	0.26 (0.23–0.29)	14,435	900 (6.2)	0.52 (0.45–0.61)	
*missing*	*2465*	*469 (19.0)*			*181*	*110 (60.8)*		*742*	170 (22.9)		*1542*	*189 (12.3)*		
Self‐report HIV status														
HIV negative	46,241	5689 (12.3)	1 (baseline)		1939	463 (23.9)	1 (baseline)	20,136	3124 (15.5)	1 (baseline)	24,166	2102 (8.7)	1 (baseline)	<0.001
HIV positive	1370	825 (60.2)	10.79 (9.14–12.74)	<0.001	164	155 (94.5)	54.90 (21.91–137.55)	611	438 (71.7)	13.79 (10.31–18.43)	595	232 (39.0)	6.71 (5.12–8.81)	
*missing*	*6892*	*2445 (35.5)*			*1936*	*1316 (68.0)*		*2693*	*855 (31.8)*		*2263*	*274 (12.1)*		
**Sisters clinic engagement**														
Clinic visits														
First visit	31,288	6508 (20.8)	1 (baseline)		2514	1395 (55.5)	1 (baseline)	13,470	3134 (23.3)	1 (baseline)	15,304	1979 (12.9)	1 (baseline)	0.07
Repeat visit	23,215	2451 (10.6)	0.45 (0.40–0.51)	<0.001	1525	539 (35.3)	0.44 (0.38–0.50)	9970	1283 (12.9)	0.49 (0.41–0.57)	11,720	629 (5.4)	0.38 (0.32–0.45)	
STI diagnosed at clinic visit														
No	35,556	4692 (13.2)	1 (baseline)		2212	993 (44.9)	1 (baseline)	13,794	2063 (15.0)	1 (baseline)	19,550	1636 (8.4)	1 (baseline)	0.01
Yes	18,947	4267 (22.5)	1.91 (1.75–2.09)	<0.001	1827	941 (51.5)	1.3 (1.05–1.61)	9646	2354 (24.4)	1.84 (1.64–2.05)	7474	972 (13.0)	1.64 (1.49–1.80)	
Visit for family planning														
No	45,053	8075 (17.9)	1 (baseline)		3616	1658 (45.9)	1 (baseline)	19,997	4080 (20.4)	1 (baseline)	21,440	2337 (10.9)	1 (baseline)	<0.001
Yes	9450	884 (9.4)	0.47 (0.36–0.61)	<0.001	423	276 (65.3)	2.22 (1.73–2.85)	3443	337 (9.8)	0.42 (0.37–0.48)	5584	271 (4.9)	0.42 (0.36–0.48)	
Testing delivery														
First programme test	39,462	8456 (21.4)	7.88 (6.62–9.38)		3560	1905 (53.5)	17.86 (11.07–28.81)	17,992	4210 (23.4)	7.73 (6.17–9.69)	17,910	2341 (13.1)	4.98 (4.08–6.09)	<0.001
Repeat programme test	15,041	503 (3.3)	1 (baseline)	<0.001	479	29 (6.1)	1 (baseline)	5448	207 (3.8)	1 (baseline)	9114	267 (2.9)	1 (baseline)	

Test positivity was lower among women either self‐reporting or testing at a Sisters clinic within the previous 6 months (2187/26,168; 8.4%) than among those who had never tested (2067/5974; 34.6%; OR 0.17 95% CI 0.16–0.18). Findings were similar for positivity among women testing in the previous 6–12 months (1531/10,808; 17.2% OR 0.31 95% CI 0.29–0.34) and >12 months (2705/9088; 29.8% OR 0.80 95% CI 0.75–0.86). This trend was the same for all time‐periods; however, in 2018–2019, test positivity among women who had tested >12 months ago was higher than positivity among women who had never tested (OR 2.02 95% CI 1.72–2.36) (Table 2).

After adjusting for age, marital status, education and urban/rural site, higher positivity remained associated with earlier time‐periods (2009–2013 vs. 2018–2019: aOR 6.08; 95% CI 5.52–6.70 and 2014–2017 vs. 2018–2019: aOR 2.15; 95% CI 2.04–2.28). After further adjusting for testing history, effect estimates decreased (2014–2017: aOR 4.03 95% CI 3.64–4.45 and 2014–2017: 1.97 95% CI 1.86–2.09) (Table 3). Similar results were obtained using the subset of data from our fully adjusted model, only with a smaller reduction in effect estimates for 2009–2013 between our crude and demographically adjusted models (OR 6.5 95% CI 5.7–7.2 to aOR 6.1 95% CI 5.5–6.7). A sensitivity analysis showed declining odds of test positivity by calendar year and the same trend with smaller effect estimates when adjusted for time since last test, in line with our findings for time‐period categories (Supplementary Table S1).

**Table 3:** Crude and adjusted logistic regression models for HIV test positivity

	Total tests	HIV‐positive tests row%	cOR (95% CI)	aOR (95% CI)^a^	aOR (95% CI)^b^
All HIV tests	54,503	8959 (16.4)	*n* = 54,503	*n* = 49,756	*n* = 47,529
Period 1: 2009–2013	4039	1934 (47.9)	8.60 (7.93–9.32)	6.08 (5.52–6.70)	4.03 (3.64–4.45)
Period 2: 2014–2017	23,440	4417 (18.8)	2.17 (2.06–2.29)	2.15 (2.04–2.28)	1.97 (1.86–2.09)
Period 3: 2018–2019	27,024	2608 (9.6)	1 (baseline)	1 (baseline)	1 (baseline)

^a^ Adjusted for demographic variables (age, marital status, education and rural/urban).

^b^ Adjusted for demographic variables and HIV testing history.

Between 2009 and 2019, 7573 women had an HIV‐negative test followed by at least one repeat HIV test at a Sisters clinic and were included in our seroconversion analysis. These women made 22,227 clinic visits and contributed 11,974 person‐years at risk (pyar). The last entry into our cohort was 19 November 2019. Median follow‐up time was 291 days (IQR 152–553) and median number of HIV tests per woman was 2 (IQR 2–3). Median time between a final negative test before a positive test among women who seroconverted was 273 days (IQR 140–529). The longest time between an HIV‐negative and an HIV‐positive test was >7 years.

A total of 464 women tested HIV positive after an initial HIV‐negative test; at a rate of 3.9 (95% CI 3.5–4.2) HIV infections per 100 pyar. Between 2009 and 2013, 36 women seroconverted at a rate of 4.2 per 100 pyar (95% CI 3.0–5.8). A further 247 women seroconverted in 2014–2017 at a rate of 3.9 per 100 pyar (95% CI 3.4–4.4) and 181 women in 2018–2019 at a rate of 3.8 per 100 pyar (95% CI 3.3–4.5).

We calculated a seroconversion rate of 4.7 per 100 pyar (95% CI 2.9–8.0) between September 2009 and May 2013. Our analysis included follow‐up data for 413 women who first tested before May 2013 but were either not included (269/413) in earlier analysis [22] because they only had one test during that period, or contributed less follow‐up time (144/413) because they later returned for subsequent tests. The seroconversion rate among these women was 1.6 per 100 pyar.

## 4 DISCUSSION

Among FSW accessing HIV testing services through the Sisters programme in Zimbabwe, we report high but declining test positivity between 2009 and 2019. Our findings suggest that this trend was mediated by an increase in more frequent individual testing both within and outside the programme. Over time, new diagnoses remained consistently high among repeat testers, at a rate between 4.2 and 3.8 per 100 pyar. Despite high seroconversion rates, the decrease seen in test positivity is likely to have been the consequence of testing saturation and increased knowledge of HIV status, which need to be factored into the interpretation of test positivity as an indicator of programme performance.

The decrease in test positivity seen at Sisters clinics is unsurprising and comparable to a decrease from 13% to 2.2% between 2000 and 2020 in non‐FSW populations across sub‐Saharan Africa [23], and 20–6% in Zimbabwe between 2011 and 2018 [24]. Although test positivity trends have not been reported for other FSW populations, similar changes were seen in HIV prevalence among women accessing FSW‐dedicated services in Kenya over a 10‐year period from 2008, which fell from 44% to 12% [25]. Our seroconversion rates were lower than 12.5 per 100 pyar (95% CI 6.9–21.2), previously reported from a subset of our data [22], due to the availability of additional follow up of women with low seroconversion rates. Estimates for our last time‐period need to be interpreted with caution as they may also be inflated and likely to become more accurate with longer follow up. Despite this, our findings reflect the minimal reduction in annual incidence seen among women 15+ years in Zimbabwe's PHIA surveys (0.5 in 2016 to 0.54 in 2020) [26, 27], and in later time‐periods are similar to rates of 3.1 and 5.3 per 100 pyar reported for young women selling sex in Zimbabwe in 2017 [28].

The HIV testing trends we observed reflect increases in testing across Zimbabwe [24]. Zimbabwe's Ministry of Health and Child Care HIV testing strategy [24, 29], UNAIDS 90‐90‐90 targets [30] and initiatives, including PEPFAR 3.0 [31], have influenced national testing coverage and targeting. Changes in World Health Organization testing guidance for key populations [6] and expansion of the Sisters programme have ensured increased testing, specifically among FSW. Resulting increases in knowledge of HIV status [21, 32, 33] leading to declines in undiagnosed HIV will reduce test positivity. Although we did not include a direct measure of knowledge of HIV status, we can infer increased knowledge from the testing expansion we observed, and from other studies in Zimbabwe [21, 32, 33]. A 2009–2011 study reported 58.2% of FSW knew their HIV‐positive status [32] compared to estimates closer to 80% in 2016 [21, 33]. Additionally, knowledge of HIV status has increased among all women of childbearing age in Zimbabwe, with over 95% of women tested in pregnancy by 2020 [5]. The rollout of pre‐exposure prophylaxis is also likely to have influenced testing trends; however, our analysis predates the widespread delivery in Zimbabwe. Higher test positivity earlier in the programme was likely due to the diagnosis of longer standing infections or women previously diagnosed. This was indicated by greater proportions of women never tested, longer periods since a previous test and more HIV‐positive tests among older women and those self‐reporting an HIV‐positive status. New infections in the programme also made up a greater proportion of HIV‐positive tests over time, further supporting these findings. Re‐diagnosis has been reported in other contexts. An analysis of provincial health records in South Africa found 51.3% of HIV‐positive tests to be previously diagnosed between 2017 and 2018 [34]. Other studies have restricted test positivity measures to newly identified HIV‐positive cases, excluding known positives from the denominator [16].

We found that HIV testing history mediated the association between time and test positivity; however, the interpretation of our findings is likely to be more complicated. A 2003–2007 US study found that testing history was associated with earlier diagnosis, but not with an HIV‐positive result, citing the potential interaction between HIV risk and testing behaviours [17]. A UK study of chlamydia testing also showed that reasons for seeking a test and individual HIV risk played a role in test positivity [19]. In our analysis, decreasing test positivity and an increasing proportion of younger women testing over time was likely to reflect reduced risk of seropositivity in younger age groups. In a Zimbabwean study among FSW, prevalence estimates were 1.5 times lower for FSW aged 18–19 years than 20–24 year olds [35]. Changing test positivity may have also been influenced by lower testing coverage in earlier years of the Sisters programme. This was seen in a study of antenatal care in Malawi, where suboptimal testing coverage led to underestimates of HIV prevalence [36].

Our study had limitations. Firstly, we used routine clinic data, introducing the potential for duplicate records and limiting the number of variables with which to explore confounding and interaction. Our analysis relied on self‐report testing history, requiring socially motivated responses to questions which may have introduced bias. The accuracy of our data improved over time as subsequent clinic visit data became available to update existing clinic records, and as observations became less reliant on self‐report. Although ultimately a strength of our analysis, this could have introduced bias and created disparity between earlier and later years. Although data were missing on demographic and test history variables, this did not appear to affect our findings. Despite adjusting for site location, we could not fully account for the changing catchment areas incorporated over time with the addition of new sites in our analysis. Mobility, transitions into and out of sex work and transfers to antenatal care and ART services, as well as testing availability through other providers, may contribute to women only receiving one HIV test at a Sisters clinic and, therefore, not included in our seroconversion analysis. Additionally, our seroconversion analysis used the midpoint between a woman's last HIV‐negative test and her first positive test as an estimated seroconversion date. This may have introduced bias in our estimates due to the length and variation in time between tests, clustering seroconversions in the middle of the reporting period and showing inaccurate declines towards the end [37], as well as ignoring the potential for seroconversion dates to be skewed towards the date of the HIV‐positive test [38]. We calculated seroconversion rates for the time‐periods used in our test positivity analysis, but may have observed different rates with alternative calendar intervals, depending on which side of a time split the estimated seroconversion date fell.

Our findings have implications for the interpretation of test positivity in tracking programme performance. Funding constraints have necessitated a drive for testing efficiencies, and higher positivity is often thought to reflect resource efficiency [9]. However, in our study, lower test positivity was driven by more frequent individual testing, which has been shown to be cost‐saving among FSWs [7, 17]. The increasing proportion of new and recent infections identified over time reflects greater awareness of HIV status and fewer re‐diagnoses, signalling a shift towards test positivity more closely approximating incident HIV infections. Testing less than every 6 months could delay HIV diagnosis or result in missed opportunities to test women who may disengage from services. Among non‐FSW populations in Kenya, more frequent testing in outpatient departments increased HIV diagnosis and reduced numbers of missed cases [18]. In Swaziland, a screening tool, including testing interval, to identify individuals at risk of being HIV positive and undiagnosed would have missed 25% of HIV‐positive cases [39].

## 5 CONCLUSIONS

Declining test positivity among FSW over time is likely to reflect changing testing patterns and demonstrate resource efficiencies. Understanding testing history and monitoring new HIV diagnoses from repeat tests could strengthen the interpretation of test positivity and provide a more nuanced understanding of programme performance. These insights are possible with routine HIV programme data and critical to informing testing delivery and ensuring we reach 95% of FSW diagnosed by 2030.

## COMPETING INTERESTS

The authors declare no competing interests.

## AUTHORS’ CONTRIBUTIONS

HSJ devised and conducted the analysis with input from BH, JRH, FMC and BR. SM, AT and STC provided support with data management and interpretation. PM and LC supported in understanding programme implementation, data collection and interpretation. HSJ wrote the manuscript with input from BH, JRH, FMC and BR and review from all authors. HSJ made reviewer revisions with input from co‐authors.

## FUNDING

The Sister's with a Voice programme has been funded by UN Population Fund (UNFPA), Global Fund to Fight AIDS, TB and Malaria (GFATM), USAID, PEPFAR and Elton John AIDS Foundation. HSJ is a PhD candidate funded by the Medical Research Council London Intercollegiate Doctoral Training Partnership (MRC‐LID).

## Supporting information


**Table S1**: Sensitivity analysis with crude and adjusted logistic regression models for HIV test positivity by visit year

## Data Availability

Data are available upon request to the Centre for Sexual Health and HIV/AIDS Research Zimbabwe.
