# Supplementary material for: Interpreting declines in HIV test positivity: an analysis of routine data from Zimbabwe's national sex work programme, 2009–2019
Source: J Int AIDS Soc. 2022 Jun 30;25(7):e25943. doi: 10.1002/jia2.25943 (PMC9247303; doi:10.1002/jia2.25943)
Supplement: Supplementary file 1 — Table S1: Sensitivity analysis with crude and adjusted logistic regression models for HIV test positivity by visit year [file JIA2-25-e25943-s001.docx]

**Supplementary Table 1:** Sensitivity Analysis with crude and adjusted logistic regression models for HIV test positivity by visit year

| Calander Year | Total Tests | HIV-positive (%) | cOR | 95% CI | aOR† | 95% CI | aOR ‡ | 95% CI |
| --- | --- | --- | --- | --- | --- | --- | --- | --- |
| All HIV Tests | **54,503** | **8,959 (16.4)** | **n=54,503** |  | **n=49,755** |  | **n=47,528** |  |
|  |  |  |  |  |  |  |  |  |
| 2009-2010 | 958 | 643 (67.1) | **19.79** | (16.97-23.07) | **-** | - | **-** | - |
| 2011 | 971 | 526 (54.2) | **11.46** | (9.96-13.18) | **10.97** | (9.24-13.03) | **6.15** | (5.13-7.38) |
| 2012 | 790 | 344 (43.5) | **7.48** | (6.41-8.72) | **6.82** | (5.79-8.04) | **4.39** | (3.71-5.20) |
| 2013 | 1,320 | 421 (31.9) | **4.54** | (3.98-5.18) | **4.45** | (3.88-5.12) | **3.25** | (2.82-3.76) |
| 2014 | 2,873 | 832 (29.0) | **3.95** | (3.58-4.36) | **4.00** | (3.60-4.43) | **3.23** | (2.89-3.60) |
| 2015 | 5,681 | 1,240 (21.8) | **2.71** | (2.49-2.95) | **2.62** | (2.40-2.86) | **2.39** | (2.18-2.61) |
| 2016 | 5,949 | 1,124 (18.9) | **2.26** | (2.07-2.46) | **2.23** | (2.04-2.43) | **2.04** | (1.86-2.23) |
| 2017 | 8,937 | 1,221 (13.7) | **1.53** | (1.41-1.66) | **1.54** | (1.41-1.67) | **1.45** | (1.33-1.59) |
| 2018 | 11,326 | 1,140 (10.1) | **1.08** | (1.00-1.18) | **1.06** | (0.98-1.16) | **1.05** | (0.96-1.14) |
| 2019 | 15,698 | 1,468 (9.4) | 1 (base) | - | 1 (base) | -- | 1 (base) | -- |

†adjusted for demographic variables (age, marital status, education, rural/urban)

‡adjusted for demographic variables and HIV testing history
